# Surgical Treatment of a Dens Invagination Type (І) in a Maxillary Lateral Incisor with 6-Year Follow-Up

**DOI:** 10.30476/DENTJODS.2020.84938.1109

**Published:** 2021-06

**Authors:** Fariborz Moazzami, Sareh Shirzadi, Mohammad Mehdi Shokouhi, Yasamin Ghahramani

**Affiliations:** 1 Dept. of Endodontic, School of Dentistry, Shiraz University of Medical Sciences, Shiraz, Iran; 2 Oral and Dental Disease Research Center, Dept. of Endodontic, School of Dentistry, Shiraz University of Medical Sciences, Shiraz, Iran

**Keywords:** Dens in Dente, Apicoectomy, Incisor

## Abstract

Dens invagination is a developmental anomaly that requires specific treatment approaches. The invagination is enamel-lined in the crown of the tooth truly negligible, and usually there is no extension on the level of the external amelocemental junction. A well ending surgical root canal treatment of an invaginated tooth with a retrograde filling is presented in this case report. Periapical radiographic examination, after 3 months and 6 years of follow-up, showed periapical healing with osseous formation .

## Introduction

Dens invaginatus (DI) is a growing anomaly, which eventuates in an enamel-lined cavity intruding into the crown or root prior to the mineralization phase [ 1]. The most acceptable etiologic theory is that DI results from an enfolding of the enamel organ (outer portion) into the dental papilla (inner portion) during tooth growth while forming a pocket.

The frequency of DI is reported to be 0.04-10% [ 2]. Its prevalence is the highest in permanent lateral incisors, central incisors, premolars, canines, and molars in a descending order [ 3]. It commonly occurs in maxilla rather than mandible, and in permanent instead of deciduous teeth [ 3]. Bilateral appearance is common in maxillary lateral incisors [ 4].

The most popular taxonomy was suggested by Oehlers [ 5 ], which depicted the anomaly in three categories:

Type I: a negligible form of enamel-lined, which does not enlarge over the amelocemental junction but arises within the limits of the crown [ 6].

Type II: an enamel-lined form attacking the root, which stands restricted as a blind sac. In this form, it is possible to be linked to the dental pulp.

Type III: this form is the one, which penetrates into the root perforating at the apical area showing a ‘second foramen’ in the apical or in the periodontal area. It does not have an instant link with pulp. The invagination might be completely lined by enamel, but frequently cementum is found lining the invagination [ 6]. Teeth with DI are prone to early caries and pulp necrosis. Several treatments related to this anomaly are recommended including endodontic therapy or surgery, combined treatment, or extraction [ 7- 8].Calcium hydroxide has been taken advantage in some cases to induce apical closure and promote repair. Sporadically, the presence of immature roots necessitates apexification [ 9- 10]. Surgical operations can be essential for some cases [ 11].

## Case Presentation

**Figure 1:**
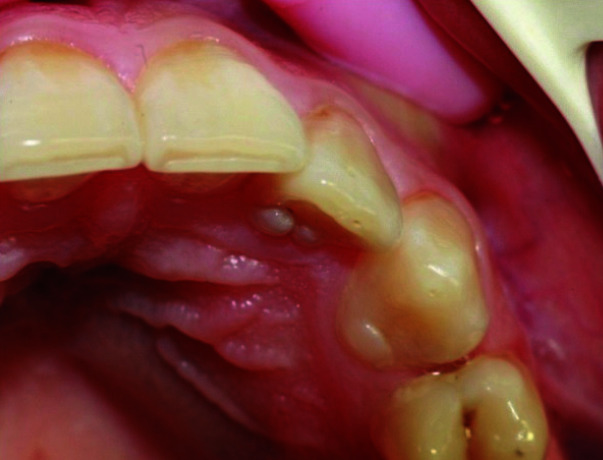
The protuberated cingulum in the palatal aspect of the left maxillary lateral incisor

**Figure 2:**
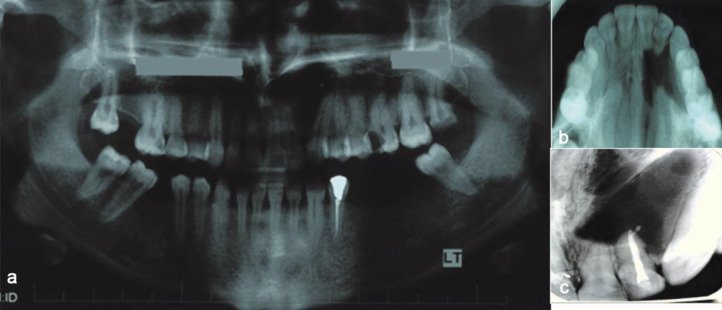
**a:** Periapical radiolucency around the apex of the laterla incisor (OPG), **b:** Periapical radiolucency around the apex of the laterla incisor (Occlusal), **c:** Obturation

A 25-year-old female patient with a history of swelling in the upper left anterior palatal region (teeth #9 to #11) referred to Endodontics Clinic affiliated to Shiraz University of Medical Sciences. Medical history was unremarkable (Figure 1). The radiographic examination showed unilocular well-defined radiolucency extended from mesial of #9 to the distal of #11. No root resorption was evident. However, root displacement was de tected (Figure 2). The tooth did not respond to thermal and electrical tests. There was no mobility, no pain on palpation, and tenderness to percussion was mild. 

A clinical diagnosis of DI (Oehlers’ Type I), necrotic pulp, and chronic apical abscess was established. The treatment plan was root canal therapy, incision, and drainage, follow-up, and apical surgery if necessary. 

The complexity of the tooth anatomy and prognosis was explained to the patient and a written consent form was obtained before each phase of the treatment.

In total, 2% lidocaine with 1:80000 epinephrine (Darupakhsh, Tehran, Iran) was injected into the buccal vestibule and clamp and rubber dam was applied. Access cavity preparation was done with high-speed turbine and diamond fissure bur (Dentsply, Maillefer, Ballaigues, Switzerland). The working length was measured with radiography (Figure 2).

Root canal was shaped with the ProTaper rotary file (DENTSPLY, Maillefer, Switzerland) and irrigated by Sodium hypo chlorite (5.25%). Due to active discharge; calcium hydroxide (Pulpdent Corp, Watertown, MA, USA) paste was used for two times and the access cavity was sealed with Cavit (3M, ESPE, Seefeld, Germany) between appointments (every 2 weeks). The root canal was obturated by lateral condensation technique with gutta-percha cones and AH-26 sealer after 4 weeks (DENTSPLY, Tulsa Dental and Tulsa, OK, USA) (Figure 2). 

Due to persistent swelling after the second month of follow-up and no change in cervical discharge, apical surgery was regarded as the treatment plan. 

**Figure 3:**
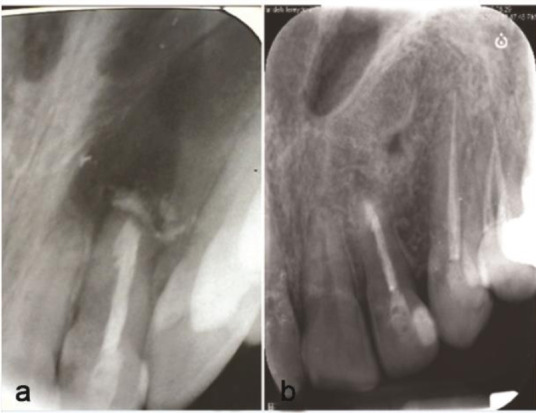
**a:** Surgical retreatment, **b:** Follow-up image after 6 years; the periapical radiolucency has disappeared

Surgical procedure was performed under surgical microscope. Treatment was initiated using 2% lidocaine with 1:80000 epinephrine (Darupakhsh, Tehran, Iran). A full-thickness mucoperiosteal triangular flap was raised following an intrasulcular incision and distal relieving incision. The lesion had perforated the cortical bone. The margins were smoothed using a round bur in a slow speed hand piece with physiological saline irrigation. Granulation tissue was removed and sent to oral pathology laboratory (Figure 3).

The root end resection (3mm) was performed with high-speed fissure bur. Cavity preparation was done, using ultrasonic device and retrograde ProRoot- MTA (Maillefer, DENTSPLY, Ballaigues, Switzerland) was inserted. The flap was repositioned and sutured. Clinical examination showed healthy clinical appearance and function after the third month. The radiographs showed partially healing of the radicular cyst, and the 6-year follow-up revealed complete healing.

## Discussion

DI must be recognized and diagnosed and treated at the soonest time in order not to produce radicular and periapical pathosis. Usually a patient cannot recognize an anomaly such as DI, until clinical signs appear, i.e., an acute dentoalveolar abscess or sinus tract [ 11]. In this report, an Oehlers' Type I invagination was on the maxillary lateral incisor, and the DI was not extended beyond the amelocemental junction. The radiograph showed unilocular well-defined radiolucency.

Interappointment medicament was considered to compensate the shortcomings of canal preparation. Calcium hydroxide was used for its antimicrobial action and for controlling the exudation of the canal. Then, the canal was obturated with lateral condensation of gutta-percha cones and AH-26 sealer after 4 weeks.

A complex procedure is required to treat invaginated teeth. A complicated root canal formation is presented in invaginated teeth, which cannot be instrumented completely. Therefore, they need to be opted for a combination of orthograde and surgical treatment [ 7, 12]. Due to persistent swelling after the second month of follow-up, apical surgery was performed. The surgery provided an additional retrograde seal with ProRoot MTA to the root canal.

Many hypotheses were suggested about the expansion of an invaginated tooth. Following to the deterioration of the dental lamina, a new theory claims that it can be led to fusion, germination, or agenesia. This is also supported by the fact that invagination is most common in maxillary lateral incisors and premolars, the most popular sites of agenesia, and that it occurs in supernumerary teeth [ 13- 14]. An informed consent was obtained from the patient. 

## Conclusion

The three-month radiograph follow-up showed partial healing of the radicular cyst and the six-year follow-up revealed complete healing.

## References

[1] Yeh SC, Lin YT, Lu SY ( 1999). Dens invaginatus in the maxillary lateral incisor: treatment of 3 cases. Oral Surg Oral Med Oral Pathol Oral Radiol Endod.

[2] Heydari A, Rahmani M ( 2015). Treatment of Dens Invagination in a Maxillary Lateral Incisor: A Case Report. Iran Endod J.

[3] Gound TG ( 1997). Dens invaginatus--a pathway to pulpal pathology: a literature review. Pract Periodontics Aesthet Dent.

[4] Shadmehr E, Farhad AR ( 2011). Clinical management of dens invaginatus type 3: a case report. Iran Endod J.

[5] Oehlers FA (1957). Dens invaginatus (dilated composite odontome). I. Variations of the invagination process and associated anterior crown forms. Oral Surg Oral Med Oral Pathol.

[6] Hulsmann M ( 1997). Dens invaginatus: aetiology, classification, prevalence, diagnosis, and treatment considerations. Int Endod J.

[7] Rotstein I, Stabholz A, Heling I, Friedman S ( 1987). Clinical considerations in the treatment of dens invaginatus. Endod Dent Traumatol.

[8] de Sousa SM, Bramante CM ( 1998). Dens invaginatus: treatment choices. Endod Dent Traumatol.

[9] Jung M ( 2004). Endodontic treatment of dens invaginatus type III with three root canals and open apical foramen. Int Endod J.

[10] Ferguson FS, Friedman S, Frazzetto V ( 1980). Successful apexification technique in an immature tooth with dens in dente. Oral Surg Oral Med Oral Pathol.

[11] Da Silva Neto UX, Hirai VH, Papalexiou V, Goncalves SB, Westphalen VP, Bramante CM, et al ( 2005). Combined endodontic therapy and surgery in the treatment of dens invaginatus Type 3: case report. J Can Dent Assoc.

[12] Froner IC, Rocha LF, da Costa WF, Barros VM, Morello D ( 1999). Complex treatment of dens invaginatus type III in maxillary lateral incisor. Endod Dent Traumatol.

[13] Tavano SM, de Sousa SM, Bramante CM ( 1994). Dens invaginatus in first mandibular premolar. Endod Dent Traumatol.

[14] Jimenez-Rubio A, Segura JJ, Jimenez-Planas A, Llamas R ( 1997). Multiple dens invaginatus affecting maxillary lateral incisors and a supernumerary tooth. Endod Dent Traumatol.

